# 1,5-Dimethyl-3-[(3-phenyl-4,5-dihydro-1,2-oxazol-5-yl)meth­yl]-1*H*-1,5-benzodiazepine-2,4(3*H*,5*H*)-dione

**DOI:** 10.1107/S1600536810042972

**Published:** 2010-10-30

**Authors:** Rachida Dardouri, Youssef Kandri Rodi, Nathalie Saffon, Lahcen El Ammari, El Mokhtar Essassi

**Affiliations:** a Laboratoire de Chimie Organique Appliquée, Faculté des Sciences et Techniques, Université Sidi Mohamed Ben Abdallah, Fés, Morocco; b Service Commun Rayons-X FR2599, Université Paul Sabatier, Bâtiment 2R1, 118 route de Narbonne, Toulouse, France; c Laboratoire de Chimie du Solide Appliquée, Faculté des Sciences, Université Mohammed V-Agdal, Avenue Ibn Battouta, BP 1014, Rabat, Morocco; d INANOTECH (Institute of Nanomaterials and Nanotechnology), Mascir, avenue de l’Armée Royale, Rabat, Morocco

## Abstract

The reaction of 3-allyl-1,5-dimethyl-1,5-benzodiazepine-2,4-dione and benzaldoxime leads to the title compound, C_21_H_21_N_3_O_3_. The mol­ecular structure is built up from two fused six- and seven-membered rings linked to a chain including a five- and six-membered ring (isoxazoline and phen­yl) *via* a methyl­ene group. The seven-membered ring displays a boat conformation. The dihedral angle between the two six-membered rings is 74.3 (1)°.

## Related literature

For the biological activity and pharmaceutical properties of benzodiazepines and their derivatives, see: Cherif Alaoui, *et al.* (2007 ▶); Fruscella *et al.* (2001 ▶); Guerrini *et al.* (2006 ▶); Jabli *et al.*, (2009 ▶); Keita *et al.* (2003 ▶); Rajarao *et al.* (2007 ▶); Kalkhambkar *et al.* (2008 ▶); Poisbeau *et al.* (1997 ▶); Smith *et al.* (1998 ▶); Kotyatkina *et al.* (2001 ▶). For their reactivity, see: Kosychova *et al.* (2004 ▶); Nabih *et al.* (2003 ▶); Reddy *et al.* (2000 ▶).
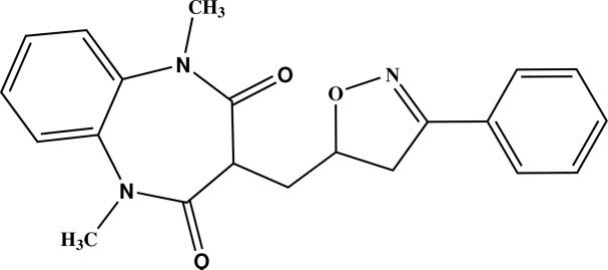


## Experimental

### 

#### Crystal data

C_21_H_21_N_3_O_3_
*M* *_r_* = 363.41
Monoclinic, 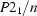

*a* = 9.3491 (2) Å
*b* = 6.9722 (1) Å
*c* = 27.9201 (5) Å
β = 93.157 (1)°
*V* = 1817.18 (6) Å^3^
*Z* = 4
Mo *K*α radiation
μ = 0.09 mm^−1^
*T* = 296 K
0.40 × 0.38 × 0.36 mm

#### Data collection

Bruker SMART CCD area-detector diffractometer
28704 measured reflections
3717 independent reflections
3261 reflections with *I* > 2σ(*I*)
*R*_int_ = 0.031

#### Refinement

*R*[*F* ^2^ > 2σ(*F* ^2^)] = 0.057
*wR*(*F* ^2^) = 0.148
*S* = 1.04
3717 reflections
246 parameters
H-atom parameters constrained
Δρ_max_ = 0.49 e Å^−3^
Δρ_min_ = −0.34 e Å^−3^

### 

Data collection: *SMART* (Bruker, 2001 ▶); cell refinement: *SAINT* (Bruker, 2001 ▶); data reduction: *SAINT*; program(s) used to solve structure: *SHELXS97* (Sheldrick, 2008 ▶); program(s) used to refine structure: *SHELXL97* (Sheldrick, 2008 ▶); molecular graphics: *ORTEP-3 for Windows* (Farrugia,1997 ▶); software used to prepare material for publication: *WinGX* (Farrugia, 1999 ▶).

## Supplementary Material

Crystal structure: contains datablocks I, global. DOI: 10.1107/S1600536810042972/im2239sup1.cif

Structure factors: contains datablocks I. DOI: 10.1107/S1600536810042972/im2239Isup2.hkl

Additional supplementary materials:  crystallographic information; 3D view; checkCIF report

## Figures and Tables

**Fig. 1.**
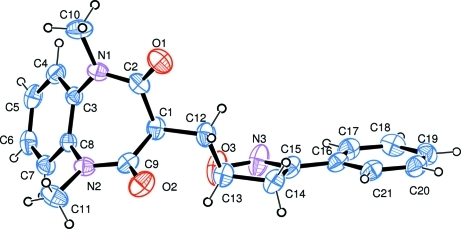
Molecular structure of the title compound with the atom-labelling scheme. Displacement ellipsoids are drawn at the 50% probability level. H atoms are represented as small circles.

## supplementary crystallographic information

### Comment

Benzodiazepines and their derivatives have attracted considerable attention from researchers due to their bioactive and pharmaceutical properties. Many members of this family are widely used as anticonvulsant, anti-anxiety, anti-seizures, analgesic, seditative, antidepressive and hypnotic or anti-inflammatory agents (Rajarao *et al.*, 2007; Guerrini *et al.*, 2006; Kotyatkina *et al.*, 2001; Fruscella *et al.*, 2001). They have also been used as antibacterial and antifungal agents (Kalkhambkar *et al.*, 2008; Smith *et al.*,1998) and in the management of skeletomuscular spasticity, panic or as premedication prior to surgery (Poisbeau *et al.*, 1997). In addition, 1,5-benzodiazepines have found applications as readily available intermediates in the synthesis of fused ring compounds such as triazolo-, oxazolo-, isoxazolo-, oxazino- or furano-benzodiazepine (Kosychova *et al.*, 2004; Nabih *et al.*, 2003; Reddy *et al.*, 2000). Benzodiazepine derivatives also find commercial use as dyes for acrylic fibers.

The search for new heterocyclic systems including the 1,5-benzodiazepine-2,4-dione moiety for biological activities therefore is of much current importance (Keita *et al.* 2003; Cherif Alaoui *et al.*, 2007; Jabli *et al.*, 2009).

In this work we were mainly interested in the reactivity of the exocyclic C=C bond of the allyl substituent towards nitriloxides. The latter are produced as intermediates from the dehydrohalogenation of benzaldoxime by a solution of sodium hypochlorite. The oxime then reacts with 3-allyl-1,5-dimethyl-1,5-benzodiazepine-2,4-dione in a biphasic medium (water-chloroform) at 0°C during 4 h to lead a unique cycloadduct 1,5-dimethyl-3-(3-phenyl-4,5-dihydro-isoxazol-5-ylmethyl)- 1,5-dihydro-benzo[*b*][1,4]diazepine-2,4-dione, in good yields (Scheme 1).

The molecular structure of 1,5-dimethyl-3-(3-phenyl-4,5-dihydro-isoxazol-5-ylmethyl)-1,5-dihydro- benzo[*b*][1,4]diazepine-2,4-dione is built up from two fused six-and seven-membered rings linked to a side-chain of a five and a six-membered ring via a methylene group (Fig.1). The isoxazoline and phenyl rings are almost coplanar with a dihedral angle between them of 2.67 (7)°. In the fused rings, the aromatic six-membered ring has a perfect planar conformation, whereas the seven-membered ring displays a boat conformation with total puckering amplitude QT = 0.999 (2) Å and spherical polar angles of θ = 76.63 (2)°, φ2 = -1.12 (1)° and φ3 = 0.83 (5)°. The torsion angles C9–C1–C12–C13 and C1–C12–C13–C14 are 72.20 (2)° and 177.20 (2)° respectively.

### Experimental

To a solution of 3-allyl-1,5-dimethyl-1,5-dihydro-benzo[*b*][1,4]diazepine-2,4-dione (0.5 g, 2 mmol) and benzaldoxime (0.3 g, 2.5 mmol) in chloroform (16 ml) was added dropwise a 24% sodium hypochlorite solution (8 ml) at 0°C. Stirring was continued for 4 h. The organic layer was dried over Na_2_SO_4_ and the solvent was evaporated under reduced pressure. The residue was then purified by column chromatography on silica gel using a mixture of hexane/ethyl acetate (*v/v* = 1/1) as eluent. Colorless crystals were isolated when the solvent was allowed to evaporate (yield: 75%).

### Refinement

H atoms were located in a difference Fourier map and treated as riding with C—H = 0.96 Å for methyl groups and C—H = 0.93 Å for all other H atoms with *U*_iso_(H) = 1.2 *U*_eq_(aromatic, methine) or *U*_iso_(H) = 1.5 *U*_eq_(methyl).

### Crystal data

**Table:**

C_21_H_21_N_3_O_3_	*F*(000) = 768
*M**_r_* = 363.41	*D*_x_ = 1.328 Mg m^−^^3^
Monoclinic, *P*2_1_/*n*	Mo *K*α radiation, λ = 0.71073 Å
Hall symbol: -P 2yn	Cell parameters from 9929 reflections
*a* = 9.3491 (2) Å	θ = 2.9–30.5°
*b* = 6.9722 (1) Å	µ = 0.09 mm^−^^1^
*c* = 27.9201 (5) Å	*T* = 296 K
β = 93.157 (1)°	Block, colourless
*V* = 1817.18 (6) Å^3^	0.40 × 0.38 × 0.36 mm
*Z* = 4	

### Data collection

**Table:**

Bruker SMART CCD area-detector diffractometer	3261 reflections with *I* > 2σ(*I*)
Radiation source: fine-focus sealed tube	*R*_int_ = 0.031
graphite	θ_max_ = 26.4°, θ_min_ = 1.5°
phi and ω scans	*h* = −11→11
28704 measured reflections	*k* = −8→8
3717 independent reflections	*l* = −34→34

### Refinement

**Table:**

Refinement on *F*^2^	Primary atom site location: structure-invariant direct methods
Least-squares matrix: full	Secondary atom site location: difference Fourier map
*R*[*F*^2^ > 2σ(*F*^2^)] = 0.057	Hydrogen site location: inferred from neighbouring sites
*wR*(*F*^2^) = 0.148	H-atom parameters constrained
*S* = 1.04	*w* = 1/[σ^2^(*F*_o_^2^) + (0.057*P*)^2^ + 1.9064*P*] where *P* = (*F*_o_^2^ + 2*F*_c_^2^)/3
3717 reflections	(Δ/σ)_max_ < 0.001
246 parameters	Δρ_max_ = 0.49 e Å^−^^3^
0 restraints	Δρ_min_ = −0.34 e Å^−^^3^

### Special details

**Table:**

Geometry. All e.s.d.'s (except the e.s.d. in the dihedral angle between two l.s. planes) are estimated using the full covariance matrix. The cell e.s.d.'s are taken into account individually in the estimation of e.s.d.'s in distances, angles and torsion angles; correlations between e.s.d.'s in cell parameters are only used when they are defined by crystal symmetry. An approximate (isotropic) treatment of cell e.s.d.'s is used for estimating e.s.d.'s involving l.s. planes.
Refinement. Refinement of *F*^2^ against ALL reflections. The weighted *R*-factor *wR* and goodness of fit *S* are based on *F*^2^, conventional *R*-factors *R* are based on *F*, with *F* set to zero for negative *F*^2^. The threshold expression of *F*^2^ > σ(*F*^2^) is used only for calculating *R*-factors(gt) *etc*. and is not relevant to the choice of reflections for refinement. *R*-factors based on *F*^2^ are statistically about twice as large as those based on *F*, and *R*- factors based on ALL data will be even larger.

### Fractional atomic coordinates and isotropic or equivalent isotropic displacement parameters (Å^2^)

**Table:**

	*x*	*y*	*z*	*U*_iso_*/*U*_eq_	
O1	0.30152 (16)	0.5636 (3)	0.20375 (6)	0.0494 (4)	
O2	0.22075 (18)	0.1345 (3)	0.12654 (6)	0.0491 (4)	
O3	0.07230 (18)	0.6193 (3)	0.04335 (7)	0.0613 (6)	
N1	0.07203 (18)	0.5003 (2)	0.22029 (6)	0.0325 (4)	
N2	0.00716 (18)	0.1746 (2)	0.15947 (6)	0.0318 (4)	
N3	0.1055 (2)	0.7758 (4)	0.01417 (8)	0.0563 (6)	
C1	0.1419 (2)	0.4598 (3)	0.13807 (7)	0.0330 (4)	
H1	0.0487	0.5157	0.1282	0.040*	
C2	0.1802 (2)	0.5157 (3)	0.18989 (8)	0.0342 (5)	
C3	−0.0714 (2)	0.4552 (3)	0.20444 (7)	0.0273 (4)	
C4	−0.1840 (2)	0.5679 (3)	0.21943 (7)	0.0346 (5)	
H4	−0.1645	0.6706	0.2400	0.042*	
C5	−0.3242 (2)	0.5290 (3)	0.20406 (8)	0.0389 (5)	
H5	−0.3982	0.6047	0.2145	0.047*	
C6	−0.3540 (2)	0.3778 (3)	0.17330 (8)	0.0382 (5)	
H6	−0.4480	0.3533	0.1624	0.046*	
C7	−0.2445 (2)	0.2627 (3)	0.15860 (7)	0.0336 (4)	
H7	−0.2655	0.1603	0.1381	0.040*	
C8	−0.1026 (2)	0.2986 (3)	0.17428 (7)	0.0268 (4)	
C9	0.1275 (2)	0.2418 (3)	0.13959 (7)	0.0334 (5)	
C10	0.1040 (3)	0.5479 (4)	0.27119 (8)	0.0460 (6)	
H10A	0.1908	0.4848	0.2824	0.069*	
H10B	0.0266	0.5058	0.2898	0.069*	
H10C	0.1155	0.6842	0.2746	0.069*	
C11	−0.0081 (3)	−0.0331 (3)	0.16577 (10)	0.0479 (6)	
H11A	0.0173	−0.0977	0.1371	0.072*	
H11B	−0.1055	−0.0626	0.1721	0.072*	
H11C	0.0540	−0.0749	0.1923	0.072*	
C12	0.2525 (2)	0.5268 (4)	0.10382 (8)	0.0395 (5)	
H12A	0.2736	0.6613	0.1097	0.047*	
H12B	0.3404	0.4548	0.1099	0.047*	
C13	0.2009 (2)	0.5008 (4)	0.05218 (8)	0.0435 (5)	
H13	0.1786	0.3656	0.0457	0.052*	
C14	0.3060 (3)	0.5754 (4)	0.01653 (8)	0.0454 (6)	
H14A	0.3993	0.6017	0.0321	0.054*	
H14B	0.3167	0.4856	−0.0096	0.054*	
C15	0.2327 (2)	0.7564 (4)	−0.00061 (8)	0.0400 (5)	
C16	0.2942 (2)	0.8996 (3)	−0.03233 (7)	0.0357 (5)	
C17	0.2153 (2)	1.0624 (4)	−0.04645 (8)	0.0416 (5)	
H17	0.1246	1.0817	−0.0352	0.050*	
C18	0.2715 (2)	1.1944 (4)	−0.07698 (9)	0.0455 (6)	
H18	0.2183	1.3021	−0.0863	0.055*	
C19	0.4069 (2)	1.1680 (4)	−0.09397 (8)	0.0441 (5)	
H19	0.4438	1.2569	−0.1148	0.053*	
C20	0.4862 (2)	1.0094 (4)	−0.07983 (8)	0.0417 (5)	
H20	0.5775	0.9920	−0.0908	0.050*	
C21	0.4301 (2)	0.8760 (3)	−0.04936 (7)	0.0376 (5)	
H21	0.4840	0.7689	−0.0401	0.045*	

### Atomic displacement parameters (Å^2^)

**Table:**

	*U*^11^	*U*^22^	*U*^33^	*U*^12^	*U*^13^	*U*^23^
O1	0.0309 (8)	0.0572 (11)	0.0589 (10)	−0.0073 (7)	−0.0071 (7)	−0.0018 (8)
O2	0.0468 (9)	0.0525 (10)	0.0488 (9)	0.0202 (8)	0.0092 (7)	−0.0012 (8)
O3	0.0354 (9)	0.0933 (15)	0.0558 (11)	0.0041 (9)	0.0070 (8)	0.0310 (10)
N1	0.0323 (8)	0.0317 (9)	0.0329 (9)	−0.0016 (7)	−0.0043 (7)	−0.0033 (7)
N2	0.0353 (9)	0.0258 (8)	0.0341 (9)	0.0033 (7)	−0.0004 (7)	−0.0021 (7)
N3	0.0358 (10)	0.0837 (17)	0.0502 (12)	0.0095 (10)	0.0098 (9)	0.0248 (12)
C1	0.0240 (9)	0.0394 (11)	0.0355 (10)	0.0023 (8)	0.0023 (8)	0.0057 (9)
C2	0.0301 (10)	0.0288 (10)	0.0433 (11)	0.0006 (8)	−0.0030 (8)	0.0018 (9)
C3	0.0294 (9)	0.0271 (9)	0.0254 (9)	−0.0012 (7)	0.0005 (7)	0.0043 (7)
C4	0.0411 (11)	0.0323 (10)	0.0311 (10)	0.0037 (9)	0.0073 (8)	0.0007 (8)
C5	0.0343 (10)	0.0454 (13)	0.0381 (11)	0.0097 (9)	0.0108 (8)	0.0105 (10)
C6	0.0268 (9)	0.0501 (13)	0.0378 (11)	−0.0031 (9)	0.0016 (8)	0.0137 (10)
C7	0.0334 (10)	0.0360 (11)	0.0309 (10)	−0.0064 (8)	−0.0022 (8)	0.0032 (8)
C8	0.0282 (9)	0.0262 (9)	0.0261 (9)	0.0003 (7)	0.0021 (7)	0.0046 (7)
C9	0.0316 (10)	0.0406 (11)	0.0279 (10)	0.0093 (9)	−0.0007 (8)	−0.0003 (8)
C10	0.0476 (13)	0.0508 (14)	0.0381 (12)	0.0017 (11)	−0.0096 (10)	−0.0118 (10)
C11	0.0525 (14)	0.0262 (11)	0.0638 (15)	0.0033 (10)	−0.0079 (12)	−0.0012 (10)
C12	0.0295 (10)	0.0477 (13)	0.0416 (12)	−0.0011 (9)	0.0047 (9)	0.0052 (10)
C13	0.0413 (12)	0.0481 (13)	0.0417 (12)	0.0002 (10)	0.0078 (10)	0.0031 (10)
C14	0.0461 (13)	0.0521 (14)	0.0388 (12)	0.0057 (11)	0.0102 (10)	0.0026 (11)
C15	0.0359 (11)	0.0525 (14)	0.0317 (10)	0.0034 (10)	0.0014 (8)	0.0006 (10)
C16	0.0353 (10)	0.0452 (12)	0.0263 (9)	0.0037 (9)	0.0004 (8)	−0.0029 (9)
C17	0.0331 (10)	0.0492 (13)	0.0426 (12)	0.0057 (10)	0.0037 (9)	−0.0041 (10)
C18	0.0430 (12)	0.0409 (13)	0.0513 (14)	0.0042 (10)	−0.0086 (10)	0.0000 (11)
C19	0.0430 (12)	0.0484 (13)	0.0404 (12)	−0.0126 (10)	−0.0015 (9)	0.0013 (10)
C20	0.0296 (10)	0.0586 (14)	0.0371 (11)	−0.0018 (10)	0.0037 (8)	−0.0073 (10)
C21	0.0347 (10)	0.0458 (12)	0.0322 (10)	0.0068 (9)	−0.0001 (8)	−0.0033 (9)

### Geometric parameters (Å, °)

**Table:**

O1—C2	1.225 (2)	C10—H10B	0.9600
O2—C9	1.220 (2)	C10—H10C	0.9600
O3—N3	1.407 (3)	C11—H11A	0.9600
O3—C13	1.469 (3)	C11—H11B	0.9600
N1—C2	1.360 (3)	C11—H11C	0.9600
N1—C3	1.424 (2)	C12—C13	1.506 (3)
N1—C10	1.474 (3)	C12—H12A	0.9700
N2—C9	1.364 (3)	C12—H12B	0.9700
N2—C8	1.420 (2)	C13—C14	1.527 (3)
N2—C11	1.467 (3)	C13—H13	0.9800
N3—C15	1.287 (3)	C14—C15	1.502 (3)
C1—C12	1.520 (3)	C14—H14A	0.9700
C1—C2	1.522 (3)	C14—H14B	0.9700
C1—C9	1.527 (3)	C15—C16	1.472 (3)
C1—H1	0.9800	C16—C21	1.391 (3)
C3—C4	1.396 (3)	C16—C17	1.398 (3)
C3—C8	1.400 (3)	C17—C18	1.378 (3)
C4—C5	1.383 (3)	C17—H17	0.9300
C4—H4	0.9300	C18—C19	1.388 (3)
C5—C6	1.378 (3)	C18—H18	0.9300
C5—H5	0.9300	C19—C20	1.377 (3)
C6—C7	1.381 (3)	C19—H19	0.9300
C6—H6	0.9300	C20—C21	1.383 (3)
C7—C8	1.397 (3)	C20—H20	0.9300
C7—H7	0.9300	C21—H21	0.9300
C10—H10A	0.9600		
			
N3—O3—C13	109.19 (16)	N2—C11—H11B	109.5
C2—N1—C3	122.92 (17)	H11A—C11—H11B	109.5
C2—N1—C10	117.75 (17)	N2—C11—H11C	109.5
C3—N1—C10	119.14 (17)	H11A—C11—H11C	109.5
C9—N2—C8	122.31 (17)	H11B—C11—H11C	109.5
C9—N2—C11	118.38 (18)	C13—C12—C1	111.85 (18)
C8—N2—C11	119.31 (18)	C13—C12—H12A	109.2
C15—N3—O3	109.9 (2)	C1—C12—H12A	109.2
C12—C1—C2	112.76 (17)	C13—C12—H12B	109.2
C12—C1—C9	112.80 (18)	C1—C12—H12B	109.2
C2—C1—C9	104.18 (16)	H12A—C12—H12B	107.9
C12—C1—H1	109.0	O3—C13—C12	108.00 (19)
C2—C1—H1	109.0	O3—C13—C14	104.44 (19)
C9—C1—H1	109.0	C12—C13—C14	113.5 (2)
O1—C2—N1	122.1 (2)	O3—C13—H13	110.2
O1—C2—C1	122.36 (19)	C12—C13—H13	110.2
N1—C2—C1	115.42 (17)	C14—C13—H13	110.2
C4—C3—C8	118.96 (18)	C15—C14—C13	101.25 (18)
C4—C3—N1	119.68 (18)	C15—C14—H14A	111.5
C8—C3—N1	121.35 (17)	C13—C14—H14A	111.5
C5—C4—C3	120.9 (2)	C15—C14—H14B	111.5
C5—C4—H4	119.6	C13—C14—H14B	111.5
C3—C4—H4	119.6	H14A—C14—H14B	109.3
C6—C5—C4	119.9 (2)	N3—C15—C16	121.3 (2)
C6—C5—H5	120.0	N3—C15—C14	113.6 (2)
C4—C5—H5	120.0	C16—C15—C14	125.12 (19)
C5—C6—C7	120.13 (19)	C21—C16—C17	118.5 (2)
C5—C6—H6	119.9	C21—C16—C15	121.3 (2)
C7—C6—H6	119.9	C17—C16—C15	120.20 (19)
C6—C7—C8	120.6 (2)	C18—C17—C16	120.2 (2)
C6—C7—H7	119.7	C18—C17—H17	119.9
C8—C7—H7	119.7	C16—C17—H17	119.9
C7—C8—C3	119.40 (18)	C17—C18—C19	120.6 (2)
C7—C8—N2	119.25 (18)	C17—C18—H18	119.7
C3—C8—N2	121.35 (17)	C19—C18—H18	119.7
O2—C9—N2	122.0 (2)	C20—C19—C18	119.7 (2)
O2—C9—C1	122.5 (2)	C20—C19—H19	120.1
N2—C9—C1	115.40 (17)	C18—C19—H19	120.1
N1—C10—H10A	109.5	C19—C20—C21	120.0 (2)
N1—C10—H10B	109.5	C19—C20—H20	120.0
H10A—C10—H10B	109.5	C21—C20—H20	120.0
N1—C10—H10C	109.5	C20—C21—C16	121.0 (2)
H10A—C10—H10C	109.5	C20—C21—H21	119.5
H10B—C10—H10C	109.5	C16—C21—H21	119.5
N2—C11—H11A	109.5		

## References

[bb1] Bruker (2001). *SAINT and *SMART** Bruker AXS Inc., Madison, Wisconsin, USA.

[bb2] Cherif Alaoui, L., Kandri Rodi, Y., Haoudi, A., Obbade, S. & Essassi, E. M. (2007). *Acta Cryst.* E**63**, o3494.

[bb3] Farrugia, L. J. (1997). *J. Appl. Cryst.* **30**, 565.

[bb4] Farrugia, L. J. (1999). *J. Appl. Cryst.* **32**, 837–838.

[bb5] Fruscella, P., Sottocorno, M., Braccio, M. D., Diomede, L., Piccardi, N., Cagnotto, A., Grossi, G., Romano, M., Mennini, T. & Roma, G. (2001). *Pharmacol. Res.* **43**, 445–452. 10.1006/phrs.2001.0800 11394936

[bb6] Guerrini, G., Costanzo, A., Ciciani, G., Bruni, F., Selleri, S., Costagli, C., Besnard, F., Costa, B., Martini, C., Siena, G. D. & Malmberg-Aiello, P. (2006). *Bioorg. Med. Chem.* **14**, 758–775. 10.1016/j.bmc.2005.08.058 16214350

[bb7] Jabli, H., Kandri Rodi, Y., Saffon, N., Essassi, E. M. & Ng, S. W. (2009). *Acta Cryst.* E**65**, o3150. 10.1107/S160053680904851X PMC2972173 21578869

[bb8] Kalkhambkar, R. G., Kulkarni, G. M., Kamanavalli, C. M., Premkumar, N., Asdaq, S. M. & Sun, C. M. (2008). *Eur. J. Med. Chem.* **43**, 2178–2188. 10.1016/j.ejmech.2007.08.007 17959273

[bb9] Keita, A., Lazrak, F., Essassi, E. M., Cherif Alaoui, I., Kandri Rodi, Y., Bellan, J. & Pierrot, M. (2003). *Phosphorus Sulfur Silicon Relat. Elem.* **178**, 1541–1548.

[bb10] Kosychova, L., Stumbreviciute, Z., Pleckaitiene, L., Janciene, R. & Puodziunaite, B. D. (2004). *Chem. Heterocycl. Compd*, **40**, 811–815.

[bb11] Kotyatkina, A. I., Zhabinsky, V. N. & Khripach Russ, V. A. (2001). *Chem. Rev.* **70**, 641–653.

[bb12] Nabih, K., Baouid, A., Hasnaoui, A., Selkti, M. & Compain, P. (2003). *New J. Chem.* **27**, 1644–1648.

[bb13] Poisbeau, P., Williams, S. R. & Mody, I. (1997). *J. Neurosci.* **17**, 3467–3475. 10.1523/JNEUROSCI.17-10-03467.1997 PMC6573704 9133372

[bb14] Rajarao, S. J., Platt, B., Sukoff, S. J., Lin, Q., Bender, C. N., Nieuwenhuijsen, B. W., Ring, R. H., Schechter, L. E., Rosenzweig-Lipson, S. & Beyer, C. E. (2007). *Neuropeptides*, **41**, 307–320. 10.1016/j.npep.2007.05.001 17637475

[bb15] Reddy, K. V. V., Rao, P. S. & Ashok, D. (2000). *Synth. Commun.* **30**, 1825–1836.

[bb16] Sheldrick, G. M. (2008). *Acta Cryst.* A**64**, 112–122. 10.1107/S0108767307043930 18156677

[bb17] Smith, R. H., Jorgen, W. L., Tirado, R. J. & Lamb, M. L. (1998). *J. Med. Chem.* **41**, 5272–5286. 10.1021/jm9804174 9857095

